# Standardized *Hydrangea serrata* (Thunb.) Ser. Extract Ameliorates Obesity in *db/db* Mice

**DOI:** 10.3390/nu13103624

**Published:** 2021-10-16

**Authors:** Hee-Soo Han, Kyung-Sook Chung, Yu-Kyoung Shin, Sun Hee Lee, Kyung-Tae Lee

**Affiliations:** 1 Department of Pharmaceutical Biochemistry, College of Pharmacy, Kyung Hee University, Seoul 02447, Korea; heesu3620@khu.ac.kr (H.-S.H.); adella76@khu.ac.kr (K.-S.C.); 2 Department of Life and Nanopharmaceutical Sciences, Graduate School, Kyung Hee University, Seoul 02447, Korea; 3 Department of New Material Development, COSMAXBIO, Seongnam 13486, Korea; ykshin@cosmax.com (Y.-K.S.); bt-shlee@cosmax.com (S.H.L.)

**Keywords:** *Hydrangea serrata* (Thunb.) Ser., obesity, *db/db*, adipogenesis, thermogenesis, gut microbiota

## Abstract

We previously reported the potential anti-obesity effects of the water extract of *Hydrangea serrata* (Thunb.) Ser. leaves (WHS) in high-fat diet-induced obese mice. As an extension of our previous study, we investigated the anti-adipogenic and anti-obesity effects of WHS and its underlying molecular mechanisms in 3T3-L1 preadipocytes and genetically obese *db/db* mice. WHS attenuated the gene expression of adipogenic transcription factors, CCAAT/enhancer binding protein (C/EBP)α, peroxisome proliferator-activated receptor (PPAR)γ, and sterol regulatory element binding protein (SREBP)-1. Moreover, WHS inhibited the mitotic clonal expansion of preadipocytes by inducing G_1_ cell cycle arrest. Oral administration of WHS alleviated body weight gain and body fat accumulation in vivo. In addition, adipocyte hypertrophy and liver steatosis were ameliorated by WHS treatment. WHS reduced C/EBPα, PPARγ, and SREBP-1 expression and activated AMPKα phosphorylation in both white adipose tissue (WAT) and liver tissue. WHS also mildly upregulated the expression of thermogenic proteins, including uncoupling protein-1, PPARs, PPARγ coactivator-1α, and sirtuin-1, in brown adipose tissue (BAT). Furthermore, WHS altered the gut microbiota composition to resemble that of wild-type mice. Taken together, our findings suggest that WHS could alleviate adiposity by inhibiting adipogenesis in WAT and the liver and modulating the gut microbiota.

## 1. Introduction

Obesity is a notable health problem worldwide, which can lead to several diseases including type 2 diabetes (T2D), non-alcoholic fatty liver disease (NAFLD), cardiovascular disease (CVD), stroke, dyslipidemia, and various types of cancer [1]. It is mainly caused by an imbalance between energy intake and expenditure, resulting from high-calorie food intake and decreased physical activity. Evidence indicates that an individual’s genetic factors also contribute to obesity [2,3]. Obesity is characterized by an excessive accumulation of body fat, which is known as white adipose tissue (WAT). The growth of WAT is mediated by two mechanisms, hyperplasia and hypertrophy, which signify the formation of new adipocytes from precursor cells and an increase in the size of mature adipocytes, respectively [4]. Therefore, regulating both the size and number of adipocytes is a key strategy for inhibiting adipogenesis and obesity.

Mitotic clonal expansion (MCE) is an important process during the differentiation of 3T3-L1 preadipocytes that mimics adipocyte hyperplasia [5]. Once cells are fully proliferated and packed, cell growth is inhibited at the G_1_ phase of the cell cycle by physical contact with neighboring cells, cells need to be fully proliferated and packed to induce adipocyte differentiation. When treated with an adipogenic hormonal cocktail containing 3-isobutyl-1-methylxanthine (IBMX), dexamethasone (DEX), and insulin, cells can re-enter the S and G_2_/M phases, which is regarded as MCE. Upon activation of insulin signaling, Akt is activated and subsequently induces downstream target protein, mammalian target of rapamycin (mTOR). The Akt/mTOR pathway plays a crucial role in adipocyte differentiation, especially in the regulation of cell cycle progression [6,7,8,9]. In contrast, the AMP-activated protein kinase (AMPK) pathway inhibits adipocyte differentiation by inducing G_1_ cell cycle arrest and suppressing mTOR phosphorylation [10]. Following MCE, early differentiation is initiated by CCAAT/enhancer-binding proteins (C/EBPs), i.e., C/EBPβ and C/EBPδ. These proteins stimulate terminal differentiation by increasing the expression of C/EBPα, peroxisome proliferator-activated receptor (PPAR)γ, and sterol regulatory element-binding protein (SREBP)-1, transcription factors that regulate lipogenic genes, including fatty acid synthase (FAS), fatty acid-binding protein 4 (FABP4), acetyl-CoA carboxylase (ACC), and stearoyl-CoA desaturase 1 (SCD1) [11]. The AMPK pathway plays a crucial role in adipogenesis by downregulating the expression of these adipogenic transcription factors and genes. Therefore, the activation of AMPK and inhibition of Akt/mTOR are both therapeutic approaches for ameliorating obesity.

WAT stores energy as triacylglycerols (TGs) and is expanded by adipogenesis, whereas brown adipose tissue (BAT) dissipates energy via thermogenesis [12]. In contrast to WAT, BAT is characterized by an abundance of mitochondria. BAT mitochondria contain a unique cellular protein, uncoupling protein-1 (UCP-1), which converts energy in the form of TGs to heat [13,14]. In BAT, PPARs are deacetylated by sirtuin-1 (SIRT-1) and interact with PPARγ coactivator-1α (PGC-1α); thus, they are involved in the regulation of lipid oxidation and thermogenesis [15,16]. Therefore, activating thermogenesis in BAT is a potential strategy for combating obesity.

Various species of *Hydrangea*, including *H. macrophylla*, *H. paniculata*, and *H. serrata*, contain several bioactive components, including coumarins, terpenoids, and iridoids [17,18,19]. *Hydrangea* extracts and their active compounds have been reported to exert protective effects against liver injury and renal injury [19,20], improve insulin resistance [21], and maintain skeletal muscle mass and function [22]. We previously examined the therapeutic effects of the water extract of *H. serrata* (Thunb.) Ser. (WHS) using high-fat diet (HFD)-induced obese mice and found that WHS may exert anti-obesity effects [23]. In addition to poor diet, genetic factors can contribute to the development of obesity [24]; thus, we further expanded our investigation on the weight loss effect of daily intake of WHS and the underlying molecular mechanisms in genetically obese *db/db* mice in this study.

## 2. Materials and Methods

### 2.1. Materials

WHS was prepared and standardized as described previously [25]. IBMX, DEX, insulin, oil red O, propidium iodide (PI), sodium orthovanadate, sodium fluoride (NaF), phenylmethylsulfonyl fluoride (PMSF), and protease inhibitor cocktail (PIC) were obtained from Sigma Aldrich Inc. (St Louis, MO, USA). Total RNA extraction kit (easy-BLUE™) and protein lysis buffer (PRO-PREP™) were purchased from iNtRON Biotechnology (Seongnam, Korea). Antibodies of ACC, p-AMPKα, AMPKα, p-Akt, p-Akt2, p-mTOR, mTOR, C/EBPα, p27, and SIRT-1 were purchased from Cell Signaling Technology Inc. (Danvers, MA, USA). Akt, cyclin D1, cyclin E, cyclin B1, CDK2, FAS, FABP4, p21, PPARα, PPARγ, UCP-1, and β-actin antibodies were purchased from Santa Cruz Biotechnology Inc. (Dallas, TX, USA). SREBP-1 antibody was obtained from Thermo Fisher Scientific Inc. (Waltham, MA, USA).

### 2.2. Cell Culture and Adipocyte Differentiation

3T3-L1 cells were cultured in growth medium (GM) supplemented with 10% bovine serum (BS) and penicillin-streptomycin (PS) (100 U/mL and 100 μg/mL). For differentiation, cells were seeded at 6 × 10^5^/cells in 6-well plates and incubated for 3 days (Day 0). After confluences, cell culture media was replaced with differentiation medium (DM) supplemented with 10% fetal bovine serum (FBS), 1% PS, and MDI (0.5 mM IBMX, 0.5 μM, and 10 μM insulin), and cells were treated with WHS (50, 100, or 200 μg/mL). After 3 days (Day 3), media was replaced with DM containing insulin (1 μM) with or without WHS every 2 days. 3T3-L1 preadipocytes were fully differentiated into adipocytes on day 9.

### 2.3. Total RNA Extraction and Quantitative Real-Time RT-PCR (qRT-PCR)

Total cellular RNA was extracted by using easy-BLUE™ kit according to the manufacturer’s instruction. The extracted RNA was amplified by RT-PCR using TOPscript™ RT DryMIX (Enzynomics Inc., Daejeon, Korea) to synthesize cDNA. Using cDNA, TB Green^®^ Premix Ex Taq™, and specific primers, the relative mRNA expression of C/EBPα, PPARγ, and SREBP-1 were quantified by QuantStudio™ 1 System (Thermo Fisher Scientific Inc., Waltham, MA, USA)

### 2.4. Protein Extraction and Western Blot

Total cellular and tissue protein were extracted as previously described [23]. The protein concentration was quantified by Bradford assay (Bio-Rad Laboratories, Inc., Hercules, CA, USA) according to the manufacturer’s instructions. Whole protein lysates (25 μg) were resolved by SDS-PAGE and electrotransferred to a PVDF membrane. The blots were incubated with specific primary and secondary antibodies. The immunoblots were detected on Amersham hyperfilm ECL (GE Healthcare Life Sciences, Chicago, IL, USA) using an ECL chemiluminescent substrate (Santa Cruz Biotechnology Inc., Dallas, TX, USA).

### 2.5. Oil Red O Staining

Oil Red O staining was conducted as described previously [23]. Briefly, 3T3-L1 cells were fully differentiated in the presence or absence of WHS as described above. The cells were washed and fixed. The fixed cells were stained with filtered oil red O solution. The Oil Red O-stained cells were observed by using an Olympus light microscope system (Tokyo, Japan). The stained intracellular lipid droplet was dissolved in isopropanol and the optical density was measured at 510 nm using a microplate reader (Molecular Devices Inc., San Jose, CA, USA).

### 2.6. PI Staining Analysis

3T3-L1 cells were incubated with DM containing MDI with or without WHS for 24 h. Cells were collected, resuspended in 100 μL PBS, fixed in 1 mL cold ethanol drop by drop, and incubated overnight. The fixed cells were then resuspended in PI staining buffer (100 μg/mL PI staining solution + 10 μg/mL RNase in PBS) and incubated for 15 min in dark. PI-stained cells were analyzed using fluorescence-activated cell sorting (FACS) cytometer (Cytomics FC 500, Beckman Coulter Inc., Brea, CA, USA).

### 2.7. Animals and Experimental Design

Six-week-old male *db/db* (C57BL/KsJ-db/db) mice and age-matched wild-type (WT; C57BL/KsJ-m+/m+) mice were obtained from the Central Lab. Animal Inc. (Seoul, Korea). Mice were acclimatized for a week under standard laboratory conditions (light/dark cycle: 12 h, 22 ± 1 °C, and 40 to 60% humidity. All animal procedures were conducted in compliance with the guidelines for Animal Care and Use of Kyung Hee University and the experimental protocol was approved by the Institutional Animal Care and Use Committee of Kyung Hee University (KHUASP-20-292). Mice were divided into 3 groups (*n* = 5): WT control group, *db/db* control group, and WHS-treated *db/db* group. All groups of mice were fed a normal chow diet and orally administered with vehicle or 300 mg/kg WHS for 5 weeks. The body weight and food intake were recorded every week during the experiment. White adipose tissues (WATs) including subcutaneous fat, inguinal fat, mesenteric fat, and perirenal fat, brown adipose tissue (BAT), and liver were removed and immediately frozen in liquid nitrogen for western blot analysis.

### 2.8. Biochemical Examination of Blood

Whole blood was collected and plasma was isolated for biochemical analysis. Aspartate transaminase (AST), alanine transaminase (ALT), blood urea nitrogen (BUN), glucose, and lipid profiles including total cholesterol (TC), triglyceride (TG), low-density lipoprotein (LDL), and high-density lipoprotein (HDL) were measured by using AU480 Chemistry Analyzer (Beckman Coulter Inc., Brea, CA, USA).

### 2.9. Histological Analysis

A part of subcutaneous fat and liver were fixed with 4% buffered formaldehyde and stained with hematoxylin and eosin (H&E) as described previously [23]. The stained slides were analyzed under a microscope (Olympus, Tokyo, Japan).

### 2.10. Microbiome Sampling and Microbiome Taxonomic Profiling (MTP)

At the end of the experiment, feces were collected from each mouse and immediately frozen in liquid nitrogen. Genomic DNA was extracted from fecal using QIAamp Fast DNA Stool Mini Kit (Qiagen, Hilden, Germany) according to the manufacturer’s instruction. PCR amplification was performed on each sample for amplifying V3–V4 regions of the bacterial 16S rRNA gene. PCR products were purified and quantified. The equal concentrations of purified products were pooled together and subjected to pyrosequencing using Illumina iSeq 100 Sequencing System (Illumina, San Diego, CA, USA). The detailed procedures were described previously [23].

### 2.11. Statistical Analysis

Data were expressed as the means ± standard deviation (SD) in vitro or standard error of the mean (SEM) in vivo, respectively. Statistical significance (*p* < 0.05) was analyzed by using Dunnett’s multiple comparison test in vitro and an unpaired Student’s *t*-test in vivo, respectively.

## 3. Results

### 3.1. WHS Inhibits the Adipogenesis and MCE of 3T3-L1 Preadipocytes

**Figure 1:**
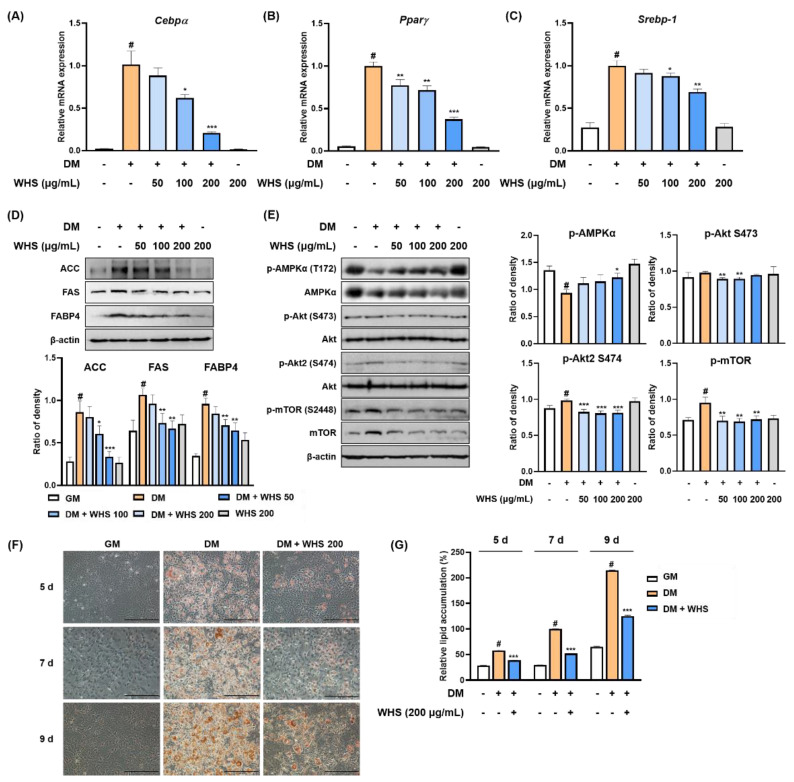
Anti-adipogenic effect of water extract of *Hydrangea serrata* (Thunb.) Ser. leaves (WHS) mediated by the regulation of the AMPK and Akt/mTOR pathways in 3T3-L1 preadipocytes. Cells were differentiated into adipocytes in the presence or absence of WHS (50, 100, or 200 μg/mL). (**A**–**C**) The mRNA expression levels of *Cebpα*, *Pparγ*, and *Srebp-1* were analyzed by qRT-PCR. (**D**,**E**) Western blot analysis was performed to determine the expression levels of ACC, FAS, and FABP4, and the activation of the AMPK and Akt/mTOR signaling pathways. (**F**) Representative microscopic images and (**G**) the quantified contents of intracellular lipid accumulation. Values are expressed as the mean ± SD. ^#^ *p* < 0.05 vs. GM control group; * *p* < 0.05, ** *p* < 0.01, and *** *p* < 0.001 vs. DM control group.

Treatment with WHS significantly reduced the mRNA expression of *Cebpα*, *Pparγ*, and *Srebp-1* (Figure 1A–C). WHS exerted anti-adipogenic effect by inhibiting the protein expression of lipogenic markers, including ACC, FAS, and FABP4 (Figure 1D). As shown in Figure 1E, the phosphorylation of AMPKα was downregulated, whereas the phosphorylation of Akt and mTOR was upregulated during adipocyte differentiation. However, treatment with WHS reversed these changes in a concentration-dependent manner. Using Oil Red O staining, we found that intracellular lipid accumulation was markedly increased over time; however, WHS treatment significantly reduced this accumulation (Figure 1F,G). These results indicated that WHS suppresses adipocyte differentiation by inhibiting *Cebpα*, *Pparγ*, and *Srebp-1* mRNA expression and ACC, FAS, and FABP4 protein expression, and regulating the AMPK and Akt/mTOR pathways.

### 3.2. WHS Inhibits MCE via G_0_/G_1_ Phase Arrest in 3T3-L1 Preadipocytes

**Figure 2:**
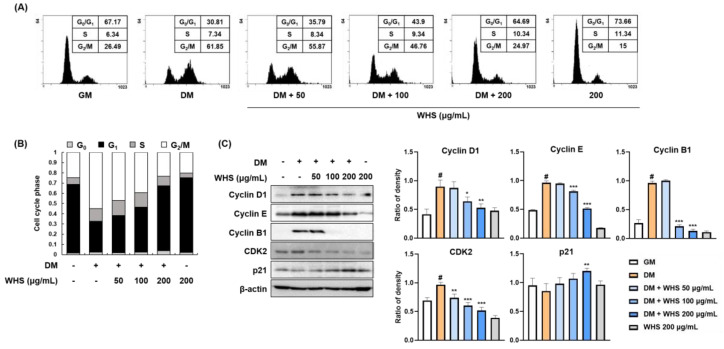
Inhibitory effect of WHS on the mitotic clonal expansion (MCE) in 3T3-L1 preadipocytes. (**A**,**B**) Flow cytometric analysis was performed to examine cell cycle progression. (**C**) The protein expression levels of cell cycle markers (cyclins, CDK2, and p21) were analyzed by western blotting. Values are expressed as the mean ± SD. ^#^ *p* < 0.05 vs. GM control group; * *p* < 0.05, ** *p* < 0.01, and *** *p* < 0.001 vs. DM control group.

In flow cytometry analysis, 3T3-L1 preadipocytes showed a larger population of G_0_/G_1_ phase cells (approximately 70% of cells) (Figure 2A,B), which was significantly decreased to approximately 30% when the cells were differentiated with DM containing MDI. In contrast, MDI treatment induced cell cycle progression to the G_2_/M phase by increasing the cell population from 26.49% to 61.85%. WHS induced G_0_/G_1_ arrest in a concentration-dependent manner by increasing the G_0_/G_1_ cell population to 35.79%, 43.90%, and 64.69% and reducing the G_2_/M cell population to 55.87%, 46.76%, and 24.97% at 50, 100, and 200 μg/mL, respectively. As shown in Figure 2C, the protein levels of cell cycle-related proteins such as cyclin D1, cyclin E, cyclin B1, and CDK2 were significantly increased in cells treated with DM containing MDI. In the presence of WHS, the MDI-induced expression of cyclins and CDK2 was decreased. Treatment with WHS abrogated the MDI-induced reduction in p21 expression. These results indicated that WHS could inhibit MCE by inducing G_0_/G_1_ arrest via regulation of cell cycle protein expression.

### 3.3. WHS Alleviates the Body Weight and Body Fat Gain of db/db Mice

**Figure 3:**
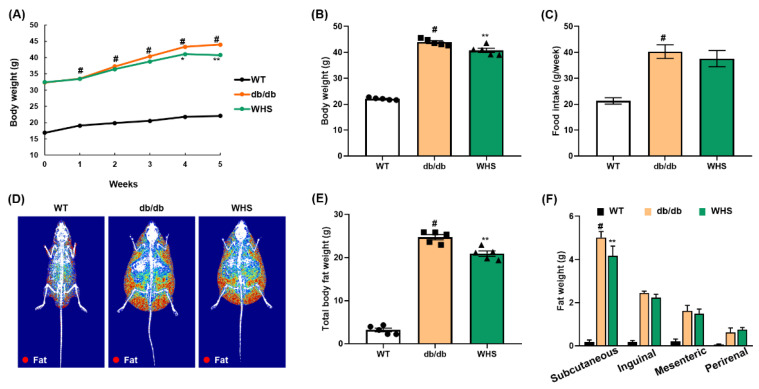
Effect of WHS on the body weight, body fat weight, and food intake of *db/db* mice. Mice (*n* = 5) were treated with vehicle or (WHS 300 mg/kg) for 5 weeks. (**A**–**C**) Body weight and food intake were measured every week. (**D**) Representative radiographic images of each group of mice analyzed by DEXA. (**E**,**F**) Total body fat weight and regional white adipose tissue (WAT) weight were measured by necropsy at the end of the experiment. Values are expressed as the mean ± SEM. ^#^ *p* < 0.05 vs. WT control group; * *p* < 0.05 and ** *p* < 0.01 vs. *db/db* control group.

A preliminary study using *db/db* mice (genetically induced obese model) shows that following the administration of 100 and 300 mg/kg WHS, 300 mg/kg was observed to be an effective dose with anti-obesity effects (Figure S1). Five-week oral administration of WHS significantly reduced weight gain without affecting food intake (Figure 3A–C). According to DEXA analysis, the body composition, total body fat distribution, and total body fat weight of WHS-treated *db/db* mice were significantly decreased (Figure 3D,E). Moreover, the body fat loss effect of WHS was correlated to a reduction in subcutaneous fat (Figure 3F). These results showed that daily administration of WHS could effectively alleviate body weight gain, which may be attributed to a reduction in subcutaneous fat weight.

### 3.4. WHS Mitigates Biochemical Parameters in Plasma of db/db Mice

We measured AST, ALT, BUN, GLU, and lipid parameters including TC, TG, LDL, and HDL in the plasma of each group of mice. As shown in Table 1, *db/db* control mice showed significantly higher levels of AST, ALT, and BUN compared with WT control mice; however, administration of WHS 300 mg/kg somewhat lowered these elevations. In addition, WHS treatment reduced the plasma levels of TC, TG, and LDL although statistical significance was not observed in TC and TG levels (Table 2). Plasma GLU levels were significantly higher in *db/db* control mice than in WT mice, which appeared to be reduced by WHS treatment (Table 2).

### 3.5. WHS Inhibits Adipocyte Hypertrophy and Adipogenesis in the WAT of db/db Mice

**Figure 4:**
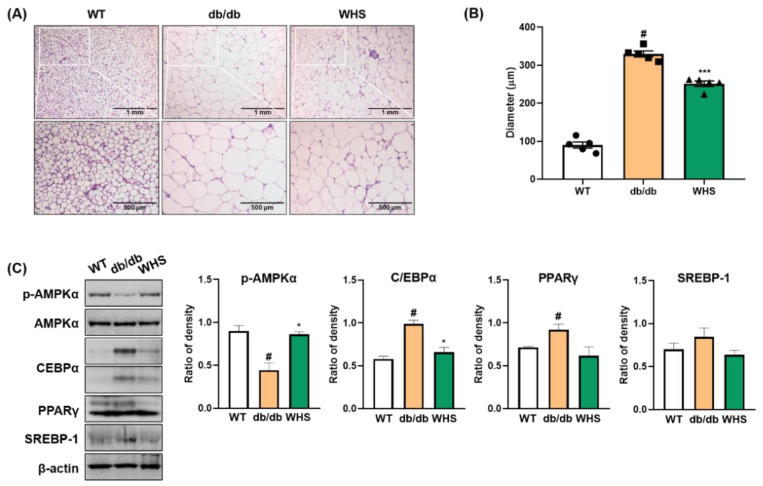
Effect of WHS on adipogenic factors in the subcutaneous fat of *db/db* mice. (**A**) Representative images of H&E-stained subcutaneous fat, (**B**) Average of adipocyte diameter. (**C**) Total protein was extracted from subcutaneous fat and western blot analysis was performed to examine the activation of AMPK and the expression of C/EBPα, PPARγ, and SREBP-1. Values are expressed as the mean ± SEM. ^#^ *p* < 0.05 vs. WT control group; * *p* < 0.05 and *** *p* < 0.001 vs. *db/db* control group.

We observed hypertrophied adipocytes (H&E-stained subcutaneous fat) in *db/db* control mice and not in WT control mice; however, oral administration of WHS reduced hypertrophy. Adipocyte diameters were measured at six arbitrarily chosen sites on each slide. The average of adipocyte diameters in *db/db* control mice (329.49 μm) was markedly larger than that in WT control mice (89.80 μm), which was significantly reduced to 251.17 μm in WHS-treated mice (Figure 4B). Consistent with the in vitro results, the protein expression levels of adipogenic transcription factors (C/EBPα, PPARγ, and SREBP-1) in the subcutaneous fat of *db/db* control mice were increased; however, WHS treatment inhibited this upregulation (Figure 4C). Moreover, WHS markedly reversed the reduced phosphorylation of AMPKα. These results demonstrated that WHS could inhibit adipocyte hypertrophy and adipogenesis by activating AMPKα and inhibiting C/EBPα, PPARγ, and SREBP-1 in the subcutaneous fat of *db/db* mice.

### 3.6. WHS Inhibits Liver Fat Accumulation in db/db Mice

**Figure 5:**
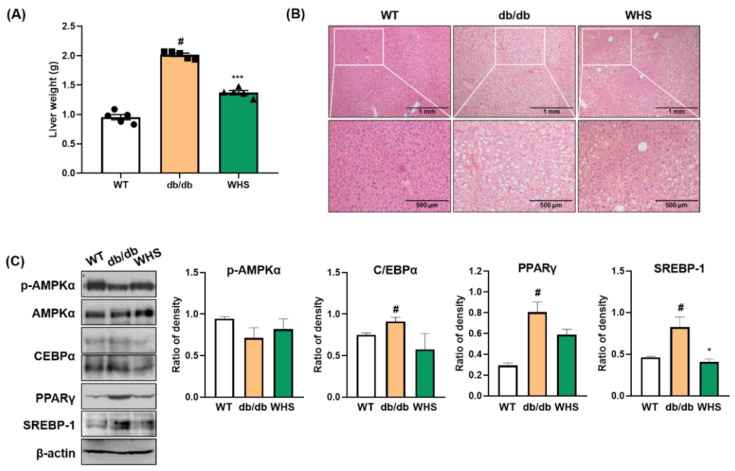
Effect of WHS on adiposity in the liver tissue of *db/db* mice. (**A**) Liver weight, (**B**) Representative images of H&E-stained liver tissue. (**C**) Total protein was extracted from the liver tissue and western blot analysis was performed to examine the activation of AMPK and the expression of C/EBPα, PPARγ, and SREBP-1. Values are expressed as the mean ± SEM. ^#^ *p* < 0.05 vs. WT control group; * *p* < 0.05 and *** *p* < 0.001 vs. *db/db* control group.

The liver weight of *db/db* control mice was markedly increased, which was significantly alleviated by WHS (Figure 5A). Microscopic analysis revealed that fat accumulation in the liver tissue of *db/db* control mice, and the color was visibly lighter than that of the liver tissue of WT control mice (Figure 5B). However, treatment with WHS reduced liver steatosis and restored redness. As fat accumulation was observed, we investigated the effect of WHS on adipogenic markers in the liver tissue. In parallel with WAT, the expression levels of C/EBPα, PPARγ, and SREBP-1 were increased, and the phosphorylation of AMPKα was decreased in *db/db* control mice; however, oral administration of WHS reversed these changes (Figure 5C). These results suggested potential preventive effects of WHS on liver steatosis by activating the AMPK pathway and inhibiting the expression of C/EBPα, PPARγ, and SREBP-1 in *db/db* mice.

### 3.7. WHS Induces Brown Fat Activation in db/db Mice

**Figure 6:**
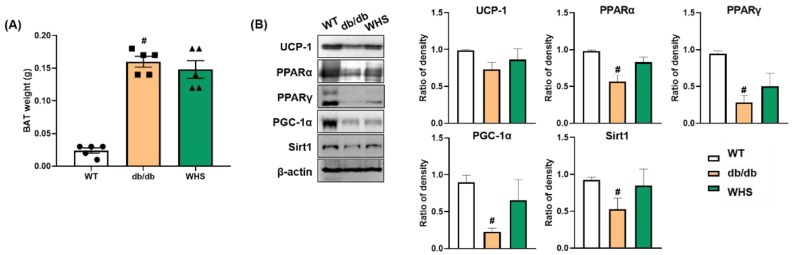
Effect of WHS on thermogenic factors in the BAT of *db/db* mice. (**A**) Brown adipose tissue (BAT) weight. (**B**) Total protein was extracted from BAT and western blot analysis was performed to examine the expression of UCP-1, PPARα, PPARγ, PGC-1α, and SIRT-1. Values are expressed as the mean ± SEM. ^#^ *p* < 0.05 vs. WT control group.

We observed differences in the protein expression of thermogenic markers; however, there was no significant change in the weight of BAT (Figure 6A). UCP-1 expression was downregulated in *db/db* control mice; however, its downregulation was abrogated by WHS treatment. The protein expression levels of PPARs (PPARα and PPARγ) and their coactivator PGC-1α were also upregulated in WHS-treated *db/db* mice (Figure 6B). SIRT-1 expression was decreased in *db/db* control mice; however, WHS reversed this effect. Overall, although statistical significance was not reached, UCP-1, PPARs, PGC-1α, and SIRT-1 expression was apparently increased in the BAT of *db/db* mice, implying potential activation of brown fat by WHS.

### 3.8. WHS Modulates the Composition of the Gut Microbiota in db/db Mice

**Figure 7:**
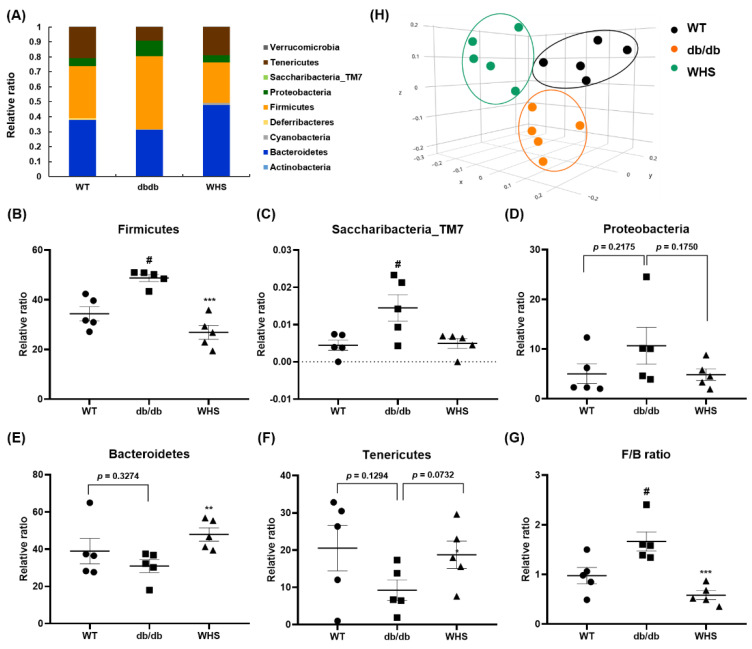
Effect of WHS on gut dysbiosis in *db/db* mice. (**A**) Gut microbiota composition of each group (phyla). (**B**–**G**) Relative ratio of the phyla *Bacteroidetes*, *Firmicutes*, *Saccharibacteria*_TM7, *Proteobacteria*, and *Tenericutes*. (**H**) β-diversity between groups analyzed by PCoA. Values are expressed as the mean ± SEM. ^#^ *p* < 0.05 vs. WT control group; ** *p* < 0.01 and *** *p* < 0.001 vs. *db/db* control group.

As shown in Figure 7A, there were differences in the microbiota composition between the groups. In *db/db* control mice, the abundance of Firmicutes, Saccharibacteria, and Proteobacteria were slightly increased, whereas the abundance of Bacteroidetes and Tenericutes were slightly decreased. These changes were abrogated in WHS-treated *db/db* mice (Figure 7B–F). The average F/B ratio of WT control mice was 1.05, indicating that the two phyla were evenly balanced. However, it was increased to 1.74 for *db/db* control mice and was decreased to 0.60 for WHS-treated mice (Figure 7G). There were considerable differences in the microbial communities between the WT control and *db/db* control groups (Figure 7H). However, the clustering of the WHS-treated group was separated from the *db/db* control group and shifted toward the WT control group. These results indicated that WHS could alleviate changes in the gut microbiota, which could contribute to obesity prevention.

## 4. Discussion

According to the World Health Organization (WHO), the prevalence of childhood obesity has greatly increased from 4% in 1975 to over 18% in 2016. As the cell number is markedly increased during the growth period, adipocyte hyperplasia mainly occurs during childhood and adolescence [26]. Once proliferated, the cell number remains constant; thus, weight gain or loss is primarily associated with the adipocyte volume in adults [27]. Depending on the number of adipocytes, overweight or obese children and adolescents are more likely to become obese in adulthood. We found that WHS effectively reduced adipocyte size in the WAT of both HFD- and genetically induced obese mice. Moreover, WHS suppressed MCE during adipocyte differentiation by arresting the cell cycle at the G_1_/S phase via the regulation of levels of cyclin D1, cyclin B1, cyclin E, CDK2, and p21. Our findings demonstrated that WHS could inhibit childhood obesity by inhibiting adipocyte hyperplasia and further prevent adult obesity.

Although obesity is mainly induced by high calorie consumption, it is also caused by various genetic factors [28]. Leptin is a hormone secreted by adipose tissue and is directly related to body fat mass. In the normal state, leptin circulates in the blood, signals satiety to the hypothalamus, and suppresses food intake, thereby reducing body weight [29]. Therefore, leptin treatment is useful for treating obesity, while congenital leptin- or leptin receptor deficiency can lead to hyperphagia and early-onset obesity [30]. Obese people show high levels of leptin due to leptin insensitivity and the lack of clinical utility of leptin, which is defined as leptin resistance [31]. We previously reported that WHS exhibited a potent weight loss effect and ameliorated leptin resistance by reducing plasma levels of leptin in HFD-induced obese mice [23]. Given that *db/db* mice are leptin receptor-deficient models that show features of obesity and leptin resistance [32], we investigated whether daily intake of WHS alleviates adiposity in genetically obese *db/db* mice in this study. We found that WHS treatment reduced body weight and body fat gain in *db/db* mice. These results indicated that WHS could ameliorate genetically induced obesity besides acquired (diet-induced) obesity.

Obesity is highly responsible for damage to multiple organs, which can lead to NAFLD induced by fat accumulation in the liver, or renal inflammation [33,34]. In this study, the *db/db* mice had significantly higher plasma levels of liver enzymes (e.g., AST and ALT) and BUN concentrations compared to wild-type mice, suggesting obesity-related liver and kidney damage [33]. Treatment of the *db/db* mice with WHS resulted in a moderate decrease in plasma AST, ALT, and BUN concentrations although statistical significance was not achieved for changes in AST and ALT levels (Table 1). Moreover, WHS treatment slightly mitigated hyperlipidemia by reducing TC, TG, and LDL levels (Table 2). Our results suggest that WHS could ameliorate obesity and obesity-associated disorders. Although the results showed no statistical significance, probably due to the short duration of administration or insufficient dosage, significant changes in the markers are expected with the long-term and/or high dose administration of WHS. The *db/db* mouse model used in this study is a well-established T2D model characterized by obesity, decreased insulin receptor sensitivity, and subsequent high levels of blood glucose (GLU) [35]. In addition to reducing GLU levels, we found that WHS upregulated adiponectin, an adipokine acts as an insulin sensitizer in WAT (Figure S2) [36]. These results suggest that WHS can effectively alleviate obesity-induced T2D.

A major strategy for treating obesity is to increase energy expenditure. Exercise is a simple method to use stored energy. Thermogenesis is another energy expenditure process, in addition to physical activity. Therefore, targeting thermogenesis is a potential approach for combating obesity [37]. Although a slight, apparent increase in the expression of BAT-related factors including UCP-1, PPARs, PGC-1α, and SIRT-1 was observed, our present study suggested no significant changes in the expression of BAT-related factors, primarily due to a relatively short duration of treatment (i.e., 12 weeks). In fact, WHS can activate AMPK, which contributes to increasing cellular NAD+ levels, activating NAD-dependent deacetylase SIRT-1 ultimately leading to PGC-1α activation, and consequently upregulating mitochondria-related genes [38,39]. Considering the activating effects of WHS on the AMPK and BAT-related genes, future studies with a longer duration of WHS therapy are warranted.

BAT is abundant in infancy; however, it regresses in adulthood, and the distribution of WAT is predominant [40]. Nevertheless, a third type of fat, referred to as beige or brite adipocytes, has been identified. Therefore, instead of BAT activation, “browning of WAT” is a possible mechanism for treating adult obesity. The browning phenomenon alters WAT metabolism from energy storage to energy utilization [41]. Increasing evidence suggests that administered agents can induce browning through several mechanisms, including UCP-1- and PGC-1α-mediated mechanisms [42], SIRT-1-mediated PPARγ and PRDM-16 deacetylation and their interaction [43], the norepinephrine-β3-adrenergic pathway [44], and the MAPK pathway [45]. In the present study, we observed the increased levels of UCP-1, PPARs, PGC-1α, and SIRT-1 in vivo. Furthermore, the activation of the p38 and ERK signaling pathways in 3T3-L1 cells following WHS treatment (Figure S3) demonstrated the potential of WHS in inducing adipocyte browning.

Increasing evidence suggests that the gut microbiota composition is affected by diet, disease state, or medications [46]. In this study, WHS-treated mice showed a gut microbiota composition that was similar to that of WT control mice, which differed from that of *db/db* control mice. Our results demonstrated that daily administration of WHS could reduce obesity by modulating the gut microbiota. Short-chain fatty acids (SCFAs), metabolites derived from bacteria, can act as signaling molecules to activate various pathways, such as the AMPK, PGC-1α, and PPAR pathways [47]. Therefore, the gut microbiota plays a crucial role in energy metabolism by producing their metabolites [48]. Bacteria members of *Bacteroidetes* mainly produce acetate and propionate, while *Firmicutes* mostly produce butyrate [49]. We found that the abundance of major genera (*Alistipes*, *Bacteroides*, and *Odoribacter*) in *Bacteroidetes* were increased in WHS-treated mice, whereas that in *Firmicutes* (*Lachnospiraceae*, *Oscillibacter*, *Roseburia*, PAC001360_g, and PAC001228_g) were decreased (Figure S4). However, these changes showed no statistical significance due to large standard error between groups and lack of samples. A variety of bacteria are involved in producing SCFAs, therefore, analyzing metabolites derived from microbiota, rather than the abundance of bacteria, is worth investigating further.

Our current study showed conflicting results regarding the effects of WHS on food intake (Figure 3 vs. Figure S1). The *db/db* mice have a mutation of the leptin receptor, resulting in loss of food intake control. Therefore, the observed lack of WHS effects on food intake might be caused by the lack of effects of leptin as a food-intake suppressor. Future confirmative studies are required to elaborate on the effects of WHS on food intake in normal mice or diet-induced obese mice. Once additional evidence is available suggesting the effects of WHS on food intake, mechanistic studies to elucidate the anti-obesity effects of WHS through evaluating direct food-intake biomarkers (e.g., GLP-1) might be needed. Lipolysis is the metabolic process through which TG in lipid droplets is hydrolyzed into glycerol and free fatty acids (FFAs). Upon stimulation, lipolysis is mainly regulated by lipases including adipocyte triglyceride lipase (ATGL), hormone-sensitive lipase (HSL), and monoacylglycerol lipase (MGL) [50]. FFAs circulate, are transported through fatty acid translocase (FAT/CD36), and are used to generate energy by mitochondrial β-oxidation using various enzymes including carnitine palmitoyltransferase 1 (CPT1) and carnitine translocase (CAT) [51]. These processes primarily occur in mitochondria of skeletal muscle which utilizes and demands high energy [52]. Therefore, it is worth further evaluating the effect of WHS on energy expenditure and mitochondrial activity in skeletal muscle.

## 5. Conclusions

Our findings show that WHS exhibits the anti-obesity action in vivo and in vitro. WHS treatment potently decreased the expression of adipogenic markers and induced G_1_ cell cycle arrest in 3T3-L1 preadipocytes. In *db/db* obese mice, WHS markedly decreased body weight and subcutaneous fat weight. Treatment with WHS slightly reduced AST, ALT, and BUN levels in plasma. WHS also mitigated hyperlipidemia by reducing plasma TC, TG, and LDL levels. In addition, WHS significantly improved obesity of *db/db* mice through downregulation of adipogenic markers in the WAT and the liver, and upregulating thermogenic factors in BAT. Taken together, these results indicate that WHS possesses anti-obesity effects by regulating adipogenesis in WAT and liver, activating BAT, and modulating gut microbiota.

## Figures and Tables

**Table 1:** The plasma AST, ALT, and BUN levels of each group.

	WT	db/db	WHS (300 mg/kg)
AST (U/L) ^a^	53.00 ± 13.47	138.80 ± 39.97 ^#^	103.80 ± 10.99
ALT (U/L) ^a^	33.20 ± 3.11	121.40 ± 56.20 ^#^	103.40 ± 13.32
BUN (mg/dl) ^a^	19.64 ± 0.52	39.58 ± 3.22 ^#^	25.60 ± 2.50 ***

^a^ Values are expressed as the mean ± SEM. ^#^ *p* < 0.05 vs. WT control group; *** *p* < 0.001 vs. *db/db* control group.

**Table 2:** The TC, TG, LDL, HDL, and GLU levels of each group.

	WT	db/db	WHS (300 mg/kg)
TC (mg/dl) ^a^	76.60 ± 5.64	126.00 ± 6.52 ^#^	102.60 ± 44.64
TG (mg/dl) ^a^	52.20 ± 4.66	86.60 ± 28.00 ^#^	56.60 ± 16.67
LDL (mg/dl) ^a^	7.80 ± 1.64	8.40 ± 1.34	6.40 ± 0.55 *
HDL (mg/dl) ^a^	56.40 ± 0.89	68.80 ± 3.11 ^#^	71.60 ± 2.70
GLU (mg/dl) ^a^	206.20 ± 103.75	645.00 ± 6.12 ^#^	600.40 ± 44.64

^a^ Values are expressed as the mean ± SEM. ^#^ *p* < 0.05 vs. WT control group; * *p* < 0.05 vs. *db/db* control group.

## Data Availability

Not applicable.

## Supplementary Materials

The following are available online at https://www.mdpi.com/article/10.3390/nu13103624/s1, Figure S1. Effect of WHS on the body weight, food intake, and body fat weight in *db/db* mice. Mice (n = 3) were treated with vehicle or (WHS 100 or 300 mg/kg) for 5 weeks. (A–C) Body weight and food intake were measured every week. (D) Representative radiographic images of each group of mice analyzed by DEXA. (E,F) Total body fat weight and regional WATs weight were measured at the end of the experiment. Values are expressed as mean ± SEM. # *p* < 0.05 vs. WT control group; * *p* < 0.05, ** *p* < 0.01, and *** *p* < 0.001 vs. *db/db* control group. Figure S2. Effect of WHS on adiponectin expression in the subcutaneous fat of *db/db* mice. (A) Total protein was extracted from subcutaneous fat and western blot analysis was performed to examine the expression of adiponectin. Values are expressed as the mean ± SEM. # *p* < 0.05 vs. WT control group. Figure S3. Effect of WHS on ERK and p38 pathways in 3T3-L1 preadipocytes. Cells were differentiated into adipocytes in the presence or absence of WHS (50, 100, or 200 μg/mL). (A) Western blot analysis was performed to determine the activation of ERK and p38 pathways. # *p* < 0.05 vs. GM control group; ** *p* < 0.01 and *** *p* < 0.001 vs. DM control group. Figure S4. Gut microbiota composition of each group (genera).
