# Supplementary material for: Standardized Hydrangea serrata (Thunb.) Ser. Extract Ameliorates Obesity in db/db Mice
Source: Nutrients. 2021 Oct 16;13(10):3624. doi: 10.3390/nu13103624 (PMC8538090; doi:10.3390/nu13103624)
Supplement: Supplementary file 1 [file nutrients-13-03624-s001.zip › nutrients-1357163-supplementary.pdf]

Supplementary materials

Supple Figure S1.

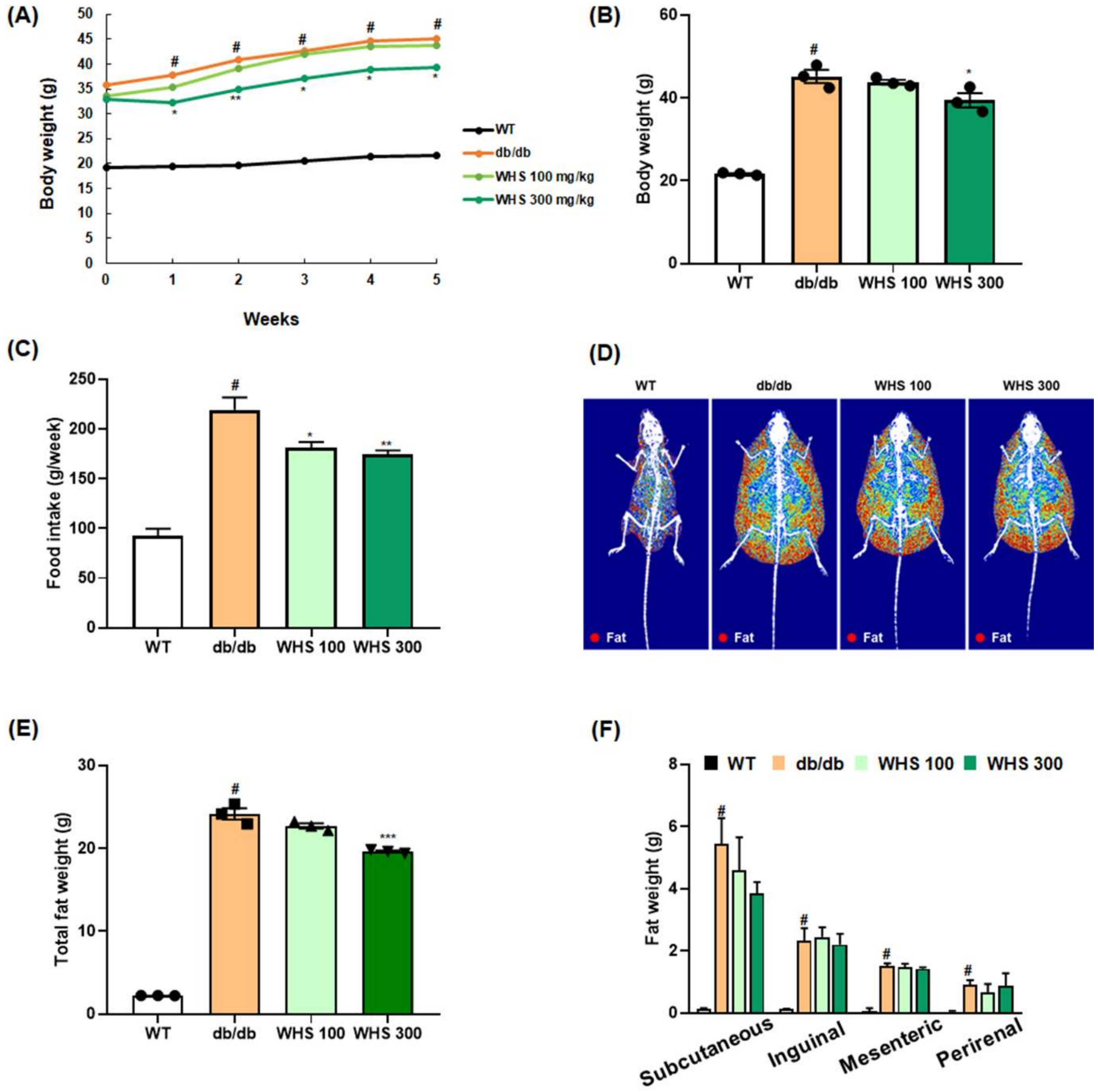

Figure S1. Effect of WHS on the body weight, food intake, and body fat weight in *db/db* mice. Mice (n = 3) were treated with vehicle or (WHS 100 or 300 mg/kg) for 5 weeks. (A-C) Body weight and food intake were measured every week. (D) Representative radiographic images of each group of mice analyzed by DEXA. (E, F) Total body fat weight and regional WATs weight were measured at the end of the experiment. Values are expressed as mean ± SEM. $^{\#}p < 0.05$ *vs.* WT control group; $^{*}p < 0.05$, $^{**}p < 0.01$, and $^{***}p < 0.001$ *vs. db/db* control group.

Supple Figure S2.

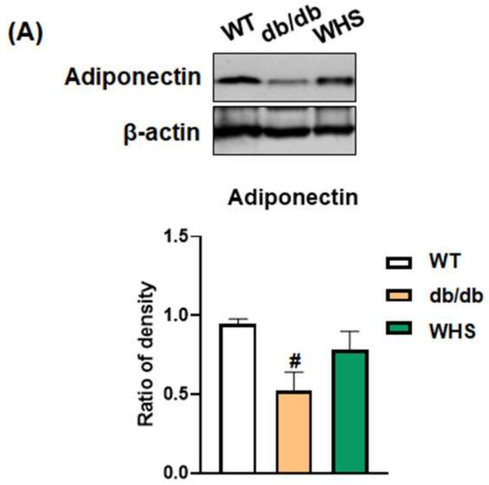

Figure S3. Effect of WHS on adiponectin expression in the subcutaneous fat of *db/db* mice. (A) Total protein was extracted from subcutaneous fat and western blot analysis was performed to examine the expression of adiponectin. Values are expressed as the mean ± SEM. $^{\#}p < 0.05$ vs. WT control group.

Supple Figure S3.

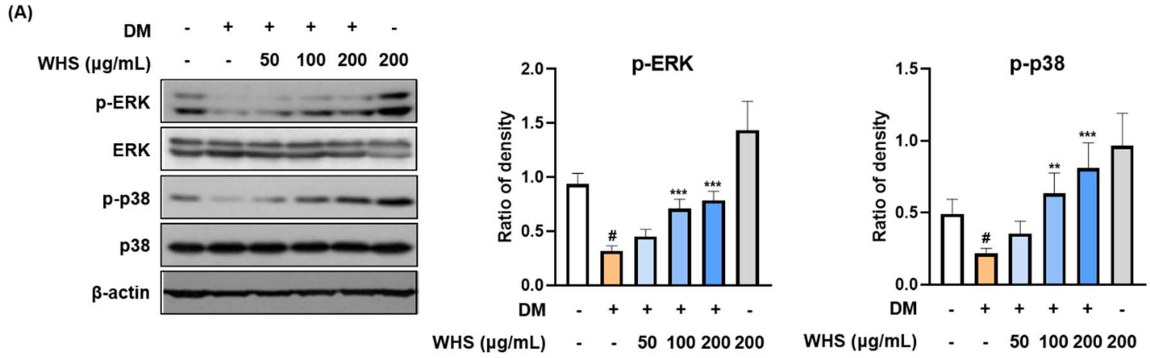

Figure S3. Effect of WHS on ERK and p38 pathways in 3T3-L1 preadipocytes. Cells were differentiated into adipocytes in the presence or absence of WHS (50, 100, or 200 μg/mL). (A) Western blot analysis was performed to determine the activation of ERK and p38 pathways. $^{\#}p < 0.05$ *vs.* GM control group; $^{**}p < 0.01$ and $^{***}p < 0.001$ *vs.* DM control group.

Supple Figure S4.

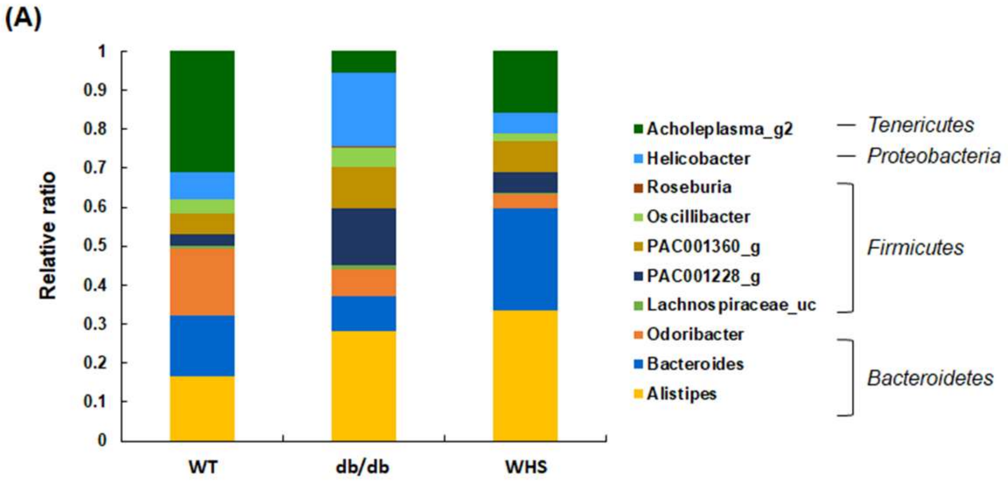

Figure S4. Gut microbiota composition of each group (genera).
